# Development and feasibility testing of the Pediatric Emergency Discharge Interaction Coding Scheme

**DOI:** 10.1111/hex.12512

**Published:** 2017-01-12

**Authors:** Janet A. Curran, Alexandra Taylor, Jill Chorney, Stephen Porter, Andrea Murphy, Shannon MacPhee, Andrea Bishop, Rebecca Haworth

**Affiliations:** ^1^ Department of Emergency Medicine IWK Health Centre Halifax NS Canada; ^2^ Faculty of Medicine Dalhousie University Halifax NS Canada; ^3^ Department of Anesthesia Dalhousie University Halifax NS Canada; ^4^ Division of Paediatric Emergency Medicine The Hospital for Sick Children Toronto ON Canada; ^5^ Department of Psychiatry & College of Pharmacy Dalhousie University Halifax NS Canada; ^6^ School of Nursing Dalhousie University Halifax NS Canada

**Keywords:** behavioural coding, discharge communication, observational study, paediatric emergency department

## Abstract

**Background:**

Discharge communication is an important aspect of high‐quality emergency care. This study addresses the gap in knowledge on how to describe discharge communication in a paediatric emergency department (ED).

**Objective:**

The objective of this feasibility study was to develop and test a coding scheme to characterize discharge communication between health‐care providers (HCPs) and caregivers who visit the ED with their children.

**Design:**

The Pediatric Emergency Discharge Interaction Coding Scheme (PEDICS) and coding manual were developed following a review of the literature and an iterative refinement process involving HCP observations, inter‐rater assessments and team consensus.

**Setting and participants:**

The coding scheme was pilot‐tested through observations of HCPs across a range of shifts in one urban paediatric ED.

**Main variables studied:**

Overall, 329 patient observations were carried out across 50 observational shifts. Inter‐rater reliability was evaluated in 16% of the observations. The final version of the PEDICS contained 41 communication elements.

**Results:**

Kappa scores were greater than .60 for the majority of communication elements. The most frequently observed communication elements were under the *Introduction* node and the least frequently observed were under the *Social Concerns* node. HCPs initiated the majority of the communication.

**Conclusion:**

Pediatric Emergency Discharge Interaction Coding Scheme addresses an important gap in the discharge communication literature. The tool is useful for mapping patterns of discharge communication between HCPs and caregivers. Results from our pilot test identified deficits in specific areas of discharge communication that could impact adherence to discharge instructions. The PEDICS would benefit from further testing with a different sample of HCPs.

## 1 Introduction

### 1.1 Background

Emergency departments (EDs) are the leading providers of unscheduled care.^1^, ^2^ Comprehensive discharge communication is a key component in the provision of quality care in these settings.^2^ With over 85% of patients discharged home from the ED, ensuring that they have the necessary information to manage their care at home after leaving the ED is vital.^2^, ^3^ However, discharge communication is often hindered by the chaotic and fast‐paced nature of the ED, which can result in frequent interruptions for health‐care providers (HCP).^4^ Other environmental barriers that impact discharge communication in the ED include overcrowding, noise, patient and caregiver stress, and time constraints.^4^, ^5^

### 1.2 Importance

Inadequate discharge communication can have undesirable consequences for the patient and family, such as underutilization of follow‐up services, adverse drug events and parental uncertainty.^3^ The effectiveness of standardized instructions to enhance discharge communication in the ED is equivocal.^6^, ^7^ The content of discharge instructions in an ED setting has been shown to vary, and there is currently no consensus on the optimal content and delivery format across different emergency practice settings and illness presentations.^8^, ^9^, ^10^, ^11^ Further, there is a lack of policy in place to support discharge communication practice in an ED context.^6^, ^12^

Patient/caregiver comprehension of discharge communication has been found to be an important factor to improve care at home and prevent unnecessary return visits.^13^ As such, greater understanding of the patterns and characteristics of discharge communication in a paediatric ED is needed to inform the design of discharge communication strategies and policies and improve outcomes for children and families. At present, there are no tools available, which could be used to characterize and study discharge communication in a paediatric ED.

### 1.3 Goals of this investigation

The aim of our pilot project was twofold. First, we sought to develop a discharge communication coding scheme and coding manual that could be used to accurately and reliably code discharge communication between HCPs and parents in a paediatric ED. Second, we conducted a pilot study to test the reliability of the coding scheme and begin to describe the content and patterns of discharge communication between paediatric emergency department HCPs and parent caregivers.

## 2 Methods

### 2.1 Development of the PEDICS

An interdisciplinary research team consisting of an ED physician, a registered nurse, a psychologist, a pharmacist and a knowledge translation researcher was established to assist with development and revision of the Pediatric Discharge Interaction Coding Scheme (PEDICS) and coding manual. Following a review of the literature, an initial list of 34 discharge communication behaviours was developed to populate the coding scheme.^3^, ^4^, ^6^, ^8^, ^13^, ^14^, ^15^, ^16^ A research assistant then shadowed three staff physicians and nurses during shifts in a paediatric ED to adjust the sequence of the discharge communication behaviours on the list to mirror how they occur in practice. The research team also grouped similar codes under parent nodes to improve the flow of the tool. We adopted a broad definition of discharge communication to include the exchange of information to inform caregivers about the diagnosis of their child, the treatments received in the ED and plan for follow‐up after discharge. Operational definitions of the codes (ie definitions based on observable characteristics) were developed for each discharge communication behaviour, including examples of what would and would not reflect the behaviour. Definitions were written using general descriptive terms that could be applied by interprofessional coders.

### 2.2 Study setting and population

A convenience sample of eight physicians and nine registered nurses working in an academic paediatric ED, with an annual census of 27 000 patients, agreed to participate in the pilot study. Data were captured during a 5‐month period of observation from June 2013 to October 2013. Trainees, such as nursing students, medical students and residents, were excluded from observations. Written informed consent was obtained from each participant. During an observation block, HCPs obtained verbal assent and consent from the patient and caregivers, respectively, before the coder entered the room.

### 2.3 Pilot testing and refinement of PEDICS

Each health‐care professional participated in a maximum of three 4‐hour observation blocks. In vivo observations were performed across a variety of shifts to capture potential differences in communication behaviour related to time of day or day of the week (eg 08:00‐12:00, 12:00‐16:00, 16:00‐20:00, 20:00‐24:00; Monday‐Sunday). The coder documented throughout the observations using the PEDICS coding sheet, noting how, what and where the discharge communication occurred. Each episode of interaction between the HCP and the caregiver was captured as a distinct interval during the 4‐hour time block. An interval was defined as a discrete interaction where communication occurred between a caregiver, patient and HCP. If the HCP left the area of communication and then returned, a new interval began. As such, the HCP could have multiple intervals for each patient and caregiver encounter.

The location where the communication occurred and the number of interruptions during each interval were recorded. It was also recorded whether the interval was the first interaction with the patient/caregiver and whether the patient was discharged during the observation block. This was performed to track the number of complete data sets of observed communication behaviours between the HCP and caregiver during a patient visit. The individual initiating the communication was also recorded. For example, when a HCP discussed a communication element with the caregiver in response to a question (RTQ), this was noted on the coding sheet. In addition to coding communication behaviour, we also collected the following demographic data for each unique patient observation: time of triage, chief complaint and diagnosis at time of discharge.

Following the initial pilot testing, a second coder was trained. The two coders contributed to refining the tool and the coding manual using an iterative process and under the supervision of the team. A second coder was present for 16% (n=8) of the observation blocks. Following every two inter‐rater observation blocks, the coders met with the research team to review any discrepancies and to make modifications to the coding manual and the coding sheet as needed. The final version of the coding scheme included 41 distinct codes, which were organized and grouped into 11 common nodes through a consensus process by the team. Each code was limited to one to three words to facilitate formatting on a single coding sheet. The communication elements were grouped into eleven categories based on the usual flow of care processes in the ED: (i) introduction, (ii) tests, (iii) medications given in the ED, (iv) discharge, (v) diagnosis, (vi) treatment plans, (vii) medications for home, (viii) social concerns, (ix) follow‐up, (x) clarification and (xi) conclusion (Table 3).

### 2.4 Analysis

Cohen's Kappa score was calculated for each of the 41 codes in the PEDICS.^17^ Descriptive statistics were calculated by collapsing intervals and communication elements into 11 nodes to explore frequency of communication behaviours. Descriptive statistics were performed using SPSS Statistics 22 (IBM SPSS Statistics for Macintosh, Version 22.0. Armonk, NY:IBM Corp.).

### 2.5 Ethics approval

The study was approved by the research ethics board at the institution where data were collected (approval #: 1014414).

## 3 Results

### 3.1 Demographics

Overall 329 patient observations were carried out across 50 observational shifts (24 physician shifts, 168 physician‐caregiver observations; 26 nurse shifts, 161 nurse‐caregiver observations). There were a total of 148 complete observations, meaning the observation included HCP and caregiver/patient interactions from admission to discharge within the 4‐hour observation block. The majority of children seen during the observation shifts were categorized as less urgent. The distribution of Canadian Triage Acuity Scale (CTAS) scores was as follows: triage level four (less urgent) (n=170, 56.1%) followed by levels three (urgent) (n=89, 29.4%) then two (emergent) (n=38, 12.5%), and the least number of children were triage levels five (non‐urgent) (n=3, 1.0%) and one (resuscitation) (n=3, 1.0%). Common presenting complaints included fever, head injury, upper and lower extremity injury, vomiting and diarrhoea, cough and abdominal pain. The triage score was not captured for 26 (8%) of the observations.

As shown in Table 1, children under the age of six comprised the largest patient population observed. The age of the patient was not recorded for 15 patients. The mother accompanied the child in 54.1% (n=85) of the nurse observations and 45% (n=75) of the physician observations (Table 2). Both parents were present in 26% (n=41) of nurse observations and 35% (n=58) of physician observations.

**Table 1:** Age groups of patients observed during nurses' and physicians' observations

Age	Physician (n=167)	Nurse (n=147)
0 to <3	63 (37.7%)	51 (34.7%)
3 to <6	26 (15.6%)	25 (17.0%)
6 to <9	31 (18.6%)	22 (15.0%)
9 to <12	17 (10.2%)	16 (10.9%)
12 to <15	22 (13.2%)	22 (15.0%)
15 to <18	8 (4.8%)	11 (7.5%)

**Table 2:** Frequency of caregivers present with patients during observations

Caregiver	Physician (n=166)	Nurse (n=157)
Mother	75 (45.2%)	85 (54.1%)
Father	26 (15.7%)	26 (16.6%)
Both parents	58 (34.9%)	41 (26.1%)
Other caregiver	7 (4.2%)	5 (3.2%)

As shown in Table 3, the most common communication elements observed for both nurses and physicians related to introduction. The least common communication elements observed for both nurses and physician were communication elements related to “social concerns.” The discharge communication behaviour of “asking whether the caregiver or patient needed clarification” was observed during 5.6% (n=9) of the nurses' observation, compared with physicians who performed this communication behaviour in 60.7% (n=102) of the patient encounters.

**Table 3:** Pediatric Emergency Discharge Interaction Coding Scheme (PEDICS) codes, definitions and the reliability of codes by Kappa score and frequency observed

	Code	Definition	Kappa^a^	Physician—patient observations^b^ (n=168)	Nurse—patient observations^b^ (n=161)
Introduction	HCP name stated	HCP states their name	.97	148 (88.1%)	99 (61.5%)
	HCP profession	HCP states their profession	.97	152 (90.5%)	95 (59.0%)
	Main concern	What brought the patient into the ED	.87	142 (84.5%)	132 (82.0%)
	Potential diagnosis	A potential new exacerbation of a chronic condition or potential cause or name for their symptoms	.63	59 (35.1%)	19 (11.8%)
	Actions needed	Generally, what will be the process of the patient's care in the ED in relation to medical care	.6	86 (51.2%)	99 (61.5%)
Tests	Diagnostic tests	Telling the patient and caregiver what diagnostic tests their child needs	.9	59 (35.1%)	27 (16.8%)
	Purpose of tests	The reason for the diagnostic test and how it is related to their child's care	.74	56 (33.3%)	20 (12.4%)
	Procedures needed	Discussing a procedure the child needs while in the ED	.25	12 (7.1%)	15 (9.3%)
	Test results	Discussing the results of the diagnostic test with the patient and caregiver	.86	68 (40.5%)	4 (2.5%)
	Test results meaning	Discussing with the patient and caregiver what the results of the diagnostic test means	.89	65 (38.7%)	1 (0.6%)
Medications given in ED	Med given ED	Administering medication in the ED	.88	3 (1.8%)	30 (18.6%)
	Name Med ED	Stating the name of the medication	1	25 (14.9%)	34 (21.1%)
	Purpose of Med ED	Educating the caregiver on how the medication will help the patient	1	25 (14.9%)	34 (21.1%)
Discharge	Review of care	Must be a second or later encounter with the patient and the HCP checking in with the patient about their condition	.37	29 (17.3%)	17 (10.6%)
	Discharged mentioned	Mentioning for the first time the potential for them to go home	.52	117 (69.6%)	21 (13.0%)
	Provide D/C form	Bringing a standardized pamphlet into the room	.85	19 (11.3%)	5 (3.1%)
	Review D/C form	Reviewing pamphlet with caregiver and child, adding new things, and asking whether they have questions	.79	17 (10.1%)	5 (3.1%)
Diagnosis	Name diagnosis	Stating a name for a new exacerbation of a chronic condition or cause or name for symptoms	.67	98 (58.3%)	1 (0.6%)
	Describe diagnosis	Describing to the child and caregiver what their diagnosis is	.67	94 (55.9%)	0 (0.0%)
	Prognosis diagnosis	Explaining to the child and caregiver what the diagnosis means, and what it means for the child's future care	.65	86 (51.2%)	0 (0.0%)
	Symptoms	Explain to the caregiver and child what they should expect to see in the progression of the diagnosis symptoms in the future	.78	84 (50%)	1 (0.6%)
Treatment plans	Treatment plan: pain	Treating the patient's pain or educating them on how their pain can be treated at home Note code this even if you coded name of medication and purpose^a^	.66	44 (26.2%)	34 (21.1%)
	Treatment plan: tests	Explaining how the caregiver will have to wait for the results of a diagnostic test that was completed in the department	.48	18 (10.7%)	5 (3.1%)
	Treatment plan: symptoms	Explaining to the caregivers how to manage their child's symptoms not including pain	.7	98 (58.3%)	12 (7.5%)
	Treatment plan: prevention	Explaining to the caregivers how to prevent this diagnosis from occurring in the future and safety education	.65	4 (2.4%)	0 (0.0%)
	Treatment plan: other	Explaining other treatment plans that are not related to pain, tests, symptoms or safety	1	6 (3.6%)	1 (0.6%)
Medication for home	Name Med 1	Stating the name of the first medication	.67	73 (43.5%)	11 (6.8%)
	Purpose Med 1	Explain to the child and caregiver the reason they were prescribed the medication and what its purpose is	.78	71 (42.3%)	7 (4.3%)
	Dose Med 1	Explain to the caregiver the dose of the medication	.55	21 (12.5%)	5 (3.1%)
	Admin Med 1	Explain to the caregiver how to give the child the medication	.85	37 (22.0%)	8 (5.0%)
	Name Med 2	Stating the name of the second medication	.55	19 (11.3%)	1 (0.6%)
	Purpose Med 2	Explain to the child and caregiver the reason they were prescribed the second medication and what its purpose is	.79	15 (8.9%)	1 (0.6%)
	Dose Med 2	Explain to the caregiver the dose of the second medication	0	5 (3.0%)	1 (0.6%)
	Admin Med 2	Explain to the caregiver how to give the child the second medication	.49	11 (6.5%)	1 (0.6%)
Social concerns	Psychosocial	Asking the caregiver whether they have any support systems at home to help with the child's care	1	6 (3.6%)	0 (0.0%)
	Socio‐economic	Asking the caregiver whether they have concerns related to the financial burden due to their insurance coverage for medications such as affording their prescriptions	0	4 (2.4%)	0 (0.0%)
Follow‐up	ED Follow‐up	Explain to the caregiver and child the reasons to return to the ED	.95	98 (58.3%)	4 (2.5%)
	PCP Follow‐up	Explain to the caregiver and child if they need to follow up with their PCP if so, when	.78	32 (19.0%)	4 (2.5%)
	Follow‐up other	Explaining to the caregiver and child the need to follow‐up with another department or physician other than the ED or PCP	.79	28 (16.7%)	3 (1.9%)
Clarification	Clarification	Asking the caregiver whether they have any further questions and to ensure they fully understand their child's care	.59	102 (60.7%)	9 (5.6%)
Conclusion	Conclusion	Conclusion of the HCP and caregiver/child relationship	.73	107 (63.7%)	12 (7.5%)

a Kappa scores: 0‐.20 as slight, .21‐.40 as fair, .41‐.60 as moderate, .61‐.80 as substantial and .81‐1 as almost perfect agreement.

b Percentage calculated based on the total number of patients observed.

Thirty of 41 codes resulted in Kappa scores between .61 and 1.0 (Table 3). In general, those communication behaviours that were less frequently observed had lower Kappa scores.

### 3.2 Location of communication

The majority of physician and nurse communication occurred in the patient's room, as shown in Table 4. During the nurse observations, 36.5% of the communication was observed during the triage process.

**Table 4:** Percentage of where the communication elements were observed in the emergency department

	Physician (%)	Nurse (%)
Patient room	96.6	51.2
Hallway	2.2	6.6
Waiting room	0.2	5.1
Triage room	0.5	36.5
Other	0.5	0.6

### 3.3 Interruptions

A total of 117 interruptions occurred during the 168 physician‐patient observations, and 33 interruptions occurred during the 161 nurse‐patient observations. The communication element “main concern” was most frequently interrupted in both physician and nurse communications, being observed 132 times and having 24 interruptions during nurses' observations and observed 142 times with 15 interruptions within physicians' observations. The second most interrupted communication element during the physicians' observations was “ED follow‐up,” being observed 98 times and had 12 interruptions.

### 3.4 Time of day

**Figure 1:**
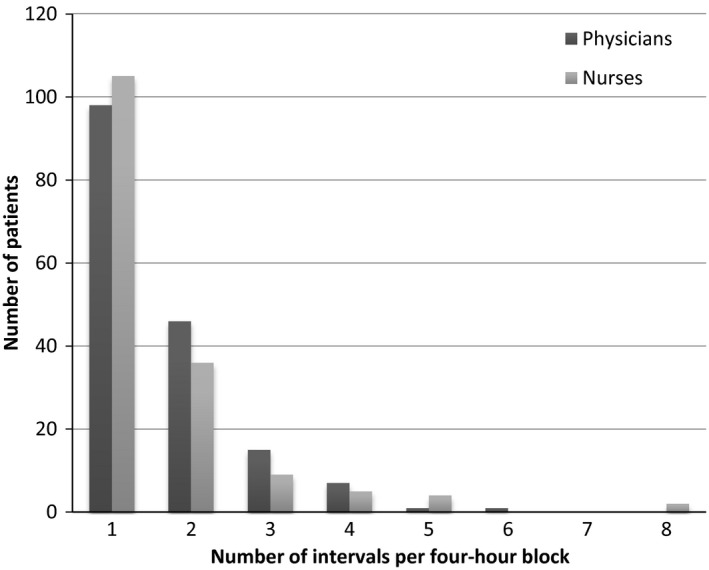
Frequency of interval (discrete interactions) per patient for a 4‐hour time block of observations

As shown in Table 5, the average number of communication intervals per patient observed during one 4‐hour observation was the highest in the late evening (20:00‐24:00) for nurses, with an average of 15.2 intervals per observation block. The highest observed communication for physicians was 15.3 intervals during the afternoon (12:00‐16:00). Figure 1 shows the frequency of intervals (discrete interactions) observed for each patient and HCP observation during a 4‐hour block. As shown in Figure 1, the majority of patients only had one discrete interaction with the HCPs during the 4‐hour time block of observations.

**Table 5:** Number of intervals based on time of day

Time of day	Average number of intervals per patient		Average number of intervals per observation block	
	Physician	Nurse	Physician	Nurse
Morning (8:00‐12:00)	1.57	1.66	7.75	9.75
Afternoon (12:00‐16:00)	1.62	1.87	15.25	8.83
Evening (16:00‐20:00)	1.71	1.98	12.45	7.6
Late Evening (20:00‐24:00)	2.0	1.67	14	15.2

### 3.5 Communication initiation

In general, the majority of the communication observed was initiated by the HCP (n=2168; 97% of elements observed with physicians, n=765; 98% of elements observed with nurses).

Although HCPs initiated most of the communication, caregivers initiated discussion about “test results” 50% of the time, primary care provider (PCP) follow‐up 25% of the time and “treatment plan/tests” 20% of the time during their interactions with nursing staff. During the physician observations, the communication element of “treatment plan: other” was initiated by the caregiver 33.3% of the time and “name of medication for discharge” 21.1% of the time.

## 4 Discussion

The development and pilot testing of the PEDICS in one urban paediatric ED with a convenience sample of 17 clinicians over four months resulted in a reliable coding scheme to map the pattern of HCP discharge communication behaviours. Many of the items with low Kappa scores were often the less frequently observed behaviours, and as such these low Kappa scores could be affected by the low base rates of these behaviours.^18^ Some of the low Kappa scores such as “Review of care” also signal the need for further clarification of definitions in the coding manual.^16^ A number of items surfaced as problematic in at least two of the coding review meetings. For example, the Kappa score for the communication element “procedures needed” was .25. This code was often mistakenly coded as “diagnostic test” by the second coder. The differences between these codes were problematic as one might be used when the HCP was telling the caregiver the child needed an X‐ray (diagnostic test) vs telling the caregiver that the child needed to be sedated to reduce a fracture based on the X‐ray results (procedure needed). The second coder in our pilot study had limited clinical experience, which was useful for revealing gaps and providing clarity to the definitions in our coding manual.

We identified a number of challenges associated with in vivo coding in a busy paediatric ED. First, the limited space and fast pace of workflow in an academic ED pose challenges for including a second coder for reliability checks. These conditions make it difficult for multiple coders to view the observation at the same time, and it was not always easy for both coders to clearly hear the exchange. Flowerdew et al.^16^ also found various sources of rater errors such as “missed behaviours” and “observed behaviours not recorded/judged” due to the high volumes of information in the ED. Previous studies in adult EDs have used audio recording to examine the clarity and content of discharge instructions.^13^, ^19^ Crain et al.^20^ also used audio recording to capture communication between HCPs and caregivers presenting to EDs and were able to establish inter‐rater reliability for verbal communication behaviours. However, none of these studies attended to non‐verbal communication behaviours, which have been identified as important features of comprehensive communication influencing patient safety and better patient adherence.^21^, ^22^ Based on the findings from our feasibility study, we suggest video recording as a data collection strategy to more accurately measure inter‐rater reliability and capture more detailed observations regarding verbal and non‐verbal behaviours of discharge communication including the influence of possible distractions, such as other children in the room or the use of android devices during communication.

Our final coding scheme included 41 codes. It is possible that this number is not feasible with in vivo coding, particularly in the early stages of development. Compared to using audio or video recordings, in vivo observations generally include simple coding schemes and therefore it is important to consider this when determining the feasibility of the number of codes.^21^ “Simple mistakes made with categorization of behaviours,” “overlapping definitions of a skill” and “misunderstandings of the definition of a skill” are also sources of inter‐rater errors. Based on discussions during our coding meetings, we reformatted the coding sheet to group common codes and used bolded text to improve the ease of identifying the nodes. Creating codes that are behavioural‐based also helps to reduce subjectivity in coding and improve inter‐rater reliability.^23^

Physician and nurse discharge communication behaviours differed in three important ways: (i) content, (ii) location and (iii) providing clarification. Although HCP communications were most commonly centred around introducing themselves, physicians discussed diagnostic tests, diagnosis, medications, treatments, discharge instructions and follow‐up information more often than nurses. Physicians most often engaged in communication with patients/caregivers in the patient's room. Similar to physicians, half of the nurse communication behaviours occurred in the patient's room; however, they also communicated in the triage area, hallways and in the waiting room. This is not surprising given the role of an ED nurse and the variety of spaces where nurses provide care and communicate with families in an ED context.^24^ Given that nurse communication occurred frequently in the triage area, it is clear that discharge communication can be initiated at the first point of contact for patients and caregivers. Lastly, despite the overall low frequency of the HCP asking the patient/caregiver whether they required any clarification, physicians were more likely to provide clarification than nurses. Checking for understanding of the information given or the need for providing clarification has been identified in the literature as an important aspect of discharge communication.^3^ PEDICS was designed to capture important discharge communication elements from a parent/caregiver perspective. Although other studies in this area do not differentiate between health disciplines,^13^, ^20^ PEDICS does allow the coder to record which HCP is initiating the communication element. This provides the opportunity to characterize discipline‐specific communication behaviours.

Findings from this feasibility study suggest the need for further qualitative work to examine HCPs' attitudes and beliefs regarding assessing caregivers social concerns in the ED. This category was added to the PEDICS after review of previous research that has highlighted the implications for low‐income families when deciding between prescribed medications or other necessities.^25^ Socio‐economic status can often be a barrier to the adherence of the discharge plan, and therefore exploring caregivers “social concerns” in the ED would allow the caregivers to voice their concerns such as limited access to primary care or being unable to afford the prescribed medication.^26^, ^27^ Ensuring that HCPs assess these types of barriers is paramount to providing tailored and relevant discharge information to all patients and caregivers.

Interestingly, this study found that HCP and patient/caregiver communication was most likely to be interrupted during two critical nodes: (i) main concern and (ii) ED follow‐up. This finding is of concern due to the importance of these communication elements for both HCP and patient/caregiver comprehension of the medical issue. Understanding “main concern” not only includes determining why the patient and caregiver came into the department, but also helps to focus the HCP on what to communicate with the patient/caregiver to ensure their expectations for the visit were met.^26^ Interruptions during these critical nodes could lead to decreased willingness on the patient/caregiver's part to follow discharge instructions.^6^, ^28^

Findings from this study also suggest that caregivers are not actively participating in the conversation by asking questions. Previous research has shown that health literacy of caregivers can impact their comprehension of written discharge communication and that HCP and patient/caregiver two‐way communication should be used to verify the caregiver understanding.^28^, ^29^, ^30^ Barriers to ensuring this exchange occurs can include a lack of patient engagement throughout the process of care, caregiver perceptions of the HCP not meeting the needs of the patient and caregiver receptivity to receiving the discharge communications.^31^, ^32^, ^33^ Additional reasons why patients and caregivers may not readily participate in two‐way communication could be a lack of knowledge regarding what questions to ask, the stress associated with their child being sick or not feeling comfortable asking questions of their HCPs.^32^ However, literature has shown that when the patient is an active participant in the discussion, greater comprehension and adherence to the treatment plan is achieved.^32^

## 5 Limitations

A number of limitations were present in this study. One limitation was the exclusion of learners as study participants. Medical and nursing students are commonly present in an academic ED. It is possible that physician discharge communication behaviour might be different in the presence of learners as learners may complete the majority of discharge communication with the caregiver. However, the primary goal of this study was to develop and evaluate a coding scheme for HCPs, and it was determined that in the context of this pilot project the inclusion of learners might influence selection of relevant discharge communication behaviours. Future work is planned to evaluate the coding scheme with different learners. During this feasibility study, the HCP was the actor of interest such that coders followed the HCP during the observation block. Patient/caregivers were not included in this exploratory work to develop the PEDICS tool, analyse the results or develop the dissemination strategy. Focusing on discharge communication from the patient's and caregiver's perspective might provide a different picture of HCP‐caregiver exchange. Our plan is to include patient/caregivers as members of the research team in the next phase of our work. The primary goal of our pilot project was to develop and evaluate a coding scheme to characterize discharge communication in an ED; therefore, we collected minimal parent/caregiver demographic data. Future research should capture more detailed parent/caregiver characteristics such as health literacy to further explore parent discharge communication behaviours. This would assist with understanding factors influencing limited parent participation in discharge communication such as identified in this pilot study.

## 6 Conclusions

Our pilot work establishes the need and usefulness of a coding scheme to characterize discharge communication in a paediatric ED setting. Analysis of inter‐rater reliability using Kappa scores found the majority of the PEDICS communication elements to have substantial inter‐rater agreement. This coding scheme is beneficial in its ability to capture the location and frequency of discrete HCP and patient/caregiver discharge communication behaviours in a paediatric ED context. Further evaluation of the PEDICS is required with a different sample of ED HCPs including learners. Findings from our feasibility study also suggest video recording as an important data collection strategy to accurately capture verbal and non‐verbal communication behaviours, strengthen inter‐rater reliability and map the multitude of factors that influence discharge communication.

## Conflict of Interest

The authors have no conflict to declare.

## Author Contributions

JAC, JC and SP conceived the study and designed the trial, and JAC obtained research funding; JAC supervised the conduct of the trial and data collection; AT completed the data collection and RH conducted inter‐rater coding; JAC, SM and AT undertook recruitment of HCPs and patients and managed the data, including quality control; JAC, JC, AM and AT provided statistical advice on study design and analysed the data; JAC and AT drafted the manuscript and all authors contributed substantially to its revision; and JAC takes responsibility for the paper as a whole.
